# Acceptability and Barriers to Use of the ASMAN Provider-Facing Electronic Platform for Peripartum Care in Public Facilities in Madhya Pradesh and Rajasthan, India: A Qualitative Study Using the Technology Acceptance Model-3

**DOI:** 10.3390/ijerph17228333

**Published:** 2020-11-11

**Authors:** Gulnoza Usmanova, Ashley Gresh, Megan A. Cohen, Young-Mi Kim, Ashish Srivastava, Chandra Shekhar Joshi, Deepak Chandra Bhatt, Rachel Haws, Rajni Wadhwa, Pompy Sridhar, Nupur Bahl, Pratibha Gaikwad, Jean Anderson

**Affiliations:** 1 Jhpiego—An Affiliate of Johns Hopkins University, New Delhi 110020, India; Gulnoza.Usmanova@Jhpiego.org (G.U.); cs.joshi@jhpiego.org (C.S.J.); deepak.bhatt@Jhpiego.org (D.C.B.); 2 Johns Hopkins University School of Nursing, Baltimore, MD 21205, USA; ashley.gresh@jhu.edu; 3 Johns Hopkins University School of Medicine, Department of Gynecology and Obstetrics, Baltimore, MD 21287, USA; Megan.a.cohen@jhu.edu (M.A.C.); janders@jhmi.edu (J.A.); 4 Jhpiego—An Affiliate of Johns Hopkins University, Baltimore, MD 21231, USA; young-mi.kim@jhpiego.org; 5 Johns Hopkins Bloomberg School of Public Health, Johns Hopkins University, Baltimore, MD 21205, USA; rachel.haws@gmail.com; 6 Project Management Unit, ASMAN: Alliance for Saving Mothers and Newborns, Mumbai 400021, India; rajni.wadhwa@reliancefoundation.org; 7 MSD for Mothers, Mumbai 4000098, India; pompy.sridhar@merck.com; 8 Reliance Foundation, Mumbai 400021, India; Nupur.Bahl@ril.com; 9 Tata Trusts, Mumbai 400005, India; pratibha@tatatrusts.org

**Keywords:** intrapartum care, maternal health, postpartum care, CDSS, mHealth, health information technology

## Abstract

The evolving field of mobile health (mHealth) is revolutionizing collection, management, and quality of clinical data in health systems. Particularly in low- and middle-income countries (LMICs), mHealth approaches for clinical decision support and record-keeping offer numerous potential advantages over paper records and in-person training and supervision. We conducted a content analysis of qualitative in-depth interviews using the Technology Acceptance Model 3 (TAM-3) to explore perspectives of providers and health managers in Madhya Pradesh and Rajasthan, India who were using the ASMAN (Alliance for Saving Mothers and Newborns) platform, a package of mHealth technologies to support management during the peripartum period. Respondents uniformly found ASMAN easy to use and felt it improved quality of care, reduced referral rates, ensured timely referral when needed, and aided reporting requirements. The TAM-3 model captured many determinants of reported respondent use behavior, including shifting workflow and job performance. However, some barriers to ASMAN digital platform use were structural and reported more often in facilities where ASMAN use was less consistent; these affect long-term impact, sustainability, and scalability of ASMAN and similar mHealth interventions. The transitioning of the program to the government, ensuring availability of dedicated funds, human resource support, and training and integration with government health information systems will ensure the sustainability of ASMAN.

## 1. Introduction

The evolving field of mobile health (mHealth) is revolutionizing the collection, management, and quality of clinical data in health systems. mHealth involves medical and public health practice supported by mobile devices, such as mobile phones, patient monitoring devices, personal digital assistants, and other wireless devices [1]. Seizing upon the promise of mobile digital platforms for data collection and dissemination, health systems are increasingly piloting, adopting, and scaling up provider-facing mHealth applications to improve health care delivery; registries and vital events tracking; electronic health records (EHRs); data collection and reporting; electronic clinical decision support systems (e-CDSSs); and provider training, education, and supportive supervision [2,3,4,5,6]. Multiple reviews have shown that mHealth approaches, particularly those that employ e-CDSSs as a central component, can lead to improvements in provider behaviors as well as patient outcomes [7,8,9,10], although few have been successfully scaled up [11]. Particularly in low- and middle-income countries (LMICs), mHealth strategies introducing e-CDSSs and EHRs offer numerous potential advantages over paper records and in-person training and supervision [12]. These include improved accessibility in remote service areas, lower costs, and the ability to aggregate and use digitized data to track progress and guide decision-making at the facility, subnational, and national levels [2,12,13]. 

Interventions that target quality of care in the intrapartum and immediate postpartum period (the peripartum period) have been identified as one of the most effective strategies for reducing maternal and neonatal deaths in LMICs [14]. In India, centralized cash transfer schemes like Janani Suraksha Yojana (JSY) have increased institutional delivery rates but failed to improve maternal and neonatal outcomes commensurately over the last decade, highlighting the need for quality improvement in peripartum care [15,16,17]. To address this need, Jhpiego, in collaboration with the Government of Rajasthan, has implemented an intrapartum and immediate postpartum quality improvement initiative based on the WHO Safe Childbirth Checklist (SCC) [18]. The results of this initiative have shown significant improvement in provider adherence to essential clinical practices, and 11% reduction in perinatal mortality [19]. As a result, the Ministry of Health and Family Welfare (MoHFW), Government of India (GoI), has scaled up this initiative throughout the country under the name Dakshata (Adroitness) [20]. However, studies of this approach have found that adherence to some essential clinical practices did not show significant improvement, and adherence to other practices declined after the intervention period [18,21,22]. 

Reviews support the promise of mHealth technologies supporting and sustaining quality improvement initiatives by increasing adherence to clinical guidelines [2], improving practitioner performance [6], strengthening peripartum care, and improving maternal and neonatal outcomes, particularly in resource-poor settings [23]. However, mHealth interventions have often languished as siloed pilot projects rather than being viewed (and evaluated) as scalable tools for overcoming health system constraints—“catalysts” for health systems strengthening [5]. Additionally, lack of attention to acceptability among health providers, an important predictor of technology adoption, has limited the success of many mHealth interventions for reproductive health [8,24]. 

### 1.1. ASMAN: A Provider-Facing mHealth Package for Peripartum Care in India

This paper focuses on a package of provider-facing mHealth technologies deployed by the ASMAN (Alliance for Saving Mothers and Newborns) platform in India to support providers’ management of mothers and neonates during the peripartum period. Initiated in 2017, the ASMAN program has aimed to reduce early maternal and newborn mortality through capacity building of providers and introduction of technology to improve quality of care in the peripartum period. The ASMAN digital platform was piloted from June 2017 to May 2020 in four districts each of Madhya Pradesh and Rajasthan states, with support from the Reliance Foundation, Tata Trusts, MSD for Mothers, the Bill and Melinda Gates Foundation, and the United States Agency for International Development (USAID). Jhpiego served as a technical implementation agency, working closely with Avalon Information Services (a digital application developer), Bodhi Health Education (gamification module developer), Edelman (communication agency), and Sambodhi Research and Communications Pvt. Ltd. (external evaluation agency). To build provider competency in intrapartum care as a prerequisite to using the ASMAN package, all intrapartum care providers at project sites attended GoI-approved Dakshata training prior to the ASMAN package roll-out. 

**Figure 1:**
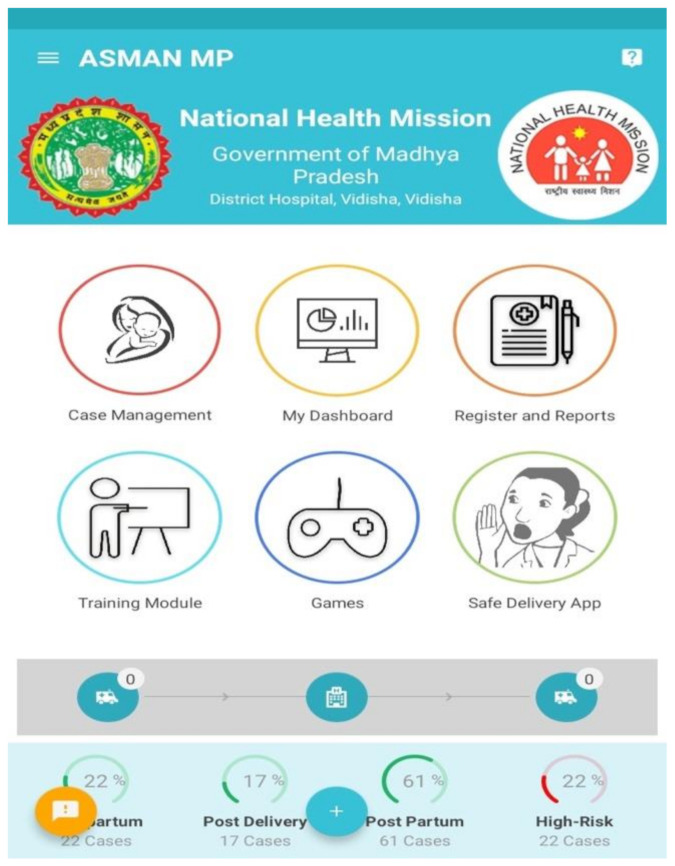
Main interface of the ASMAN (Alliance for Saving Mothers and Newborns) platform.

The ASMAN platform facilitates timely and correct clinical decision-making by providers at project sites. Designed for use by health providers, the ASMAN platform runs on a tablet stationed in registration, triage, the labor room, and postpartum areas of health facilities, and includes the following components (see Figure 1):

- Case management: A digitized case sheet is used from admission until discharge with integrated clinical rules (admission notes, e-partograph, Safe Childbirth Checklist, delivery notes, post-delivery monitoring, post-natal care, discharge slip, referral slip, events section, alerts and notifications).
- Dashboards and reports: System-generated dashboards and reports allow data in ASMAN to be reported to respective health facilities, districts, and state level managers.
- E-learning content: All GoI training modules, guidelines, and tutorials are included in the ASMAN platform and were available in audio, video, or written formats in English or in Hindi.
- ASMAN Complication Management Game: This case-based game is intended to improve management of intrapartum and immediate postpartum/postnatal complications for developing the critical thinking skills of health workers around safe childbirth.
- Safe Delivery App: Developed by the Maternity Foundation, the University of Southern Denmark, and the University of Copenhagen, this feature within the ASMAN platform gives users instant access to up-to-date clinical guidelines on basic emergency obstetrics and neonatal care.
- Remote support center: Staffed 24/7 by obstetricians and senior nurses at the district referral hospital, the support center provides guidance in cases of unclear management. Staff at the remote support system have access to all cases where expert opinion was sought.

Rajasthan and Madhya Pradesh were strategically chosen for the proof-of-concept rollout of the ASMAN program based on recommendations from India’s Health Ministry and their comparatively poor neonatal and maternal outcomes compared to the Indian average. Neonatal mortality rates in Rajasthan and Madhya Pradesh are 28 and 32 per 1000 live births, respectively—higher than the national average of 24 [25]. The maternal mortality ratio (MMR) per 100,000 live births is 164 in Rajasthan and 173 in Madhya Pradesh, higher than the national MMR of 113 in 2016–18 [26]. 

The ASMAN program was implemented at 81 facilities across four districts each of Madhya Pradesh (Jabalpur, Khargone, Ratlam, and Vidisha) and Rajasthan (Ajmer, Bhilwara, Kota, and Jhalawar). The respective state governments were consulted to select specific intervention districts that had higher neonatal and maternal mortality rates than the state average. Within those districts, government health facilities were selected that had a relatively high case load of 50 or more deliveries per month, and therefore greater need for intervention. 

### 1.2. Research Aims

New technologies face challenges being accepted in health care settings and integrated into the preexisting culture [27]. Indeed, supervision and program monitoring activities have revealed that uptake and utilization of ASMAN has not been uniform across facilities. Identifying factors that influence the adoption of the ASMAN digital platform is essential to ensuring their acceptability, effectiveness, scalability, and sustainability as the program goes to scale. A better understanding of these factors will also fill a critical information gap for designers of other multimodal health information technology platforms. 

We conducted a qualitative study to explore the experiences and perspectives of providers and health managers who use the ASMAN digital platform. Guided by the Technology Acceptance Model 3 (TAM-3), we aimed to document health providers’ and health managers’ experiences, including perceived ease of use, perceived usefulness, use intentions, and use experience with different components of the ASMAN application. We also aimed to compare differences in health providers’ and health managers’ perceptions and experiences in facilities with high versus low utilization of the ASMAN platform.

## 2. Methods 

### 2.1. Conceptualizing Technology Acceptance and Adoption: The TAM-3 Model 

**Figure 2:**
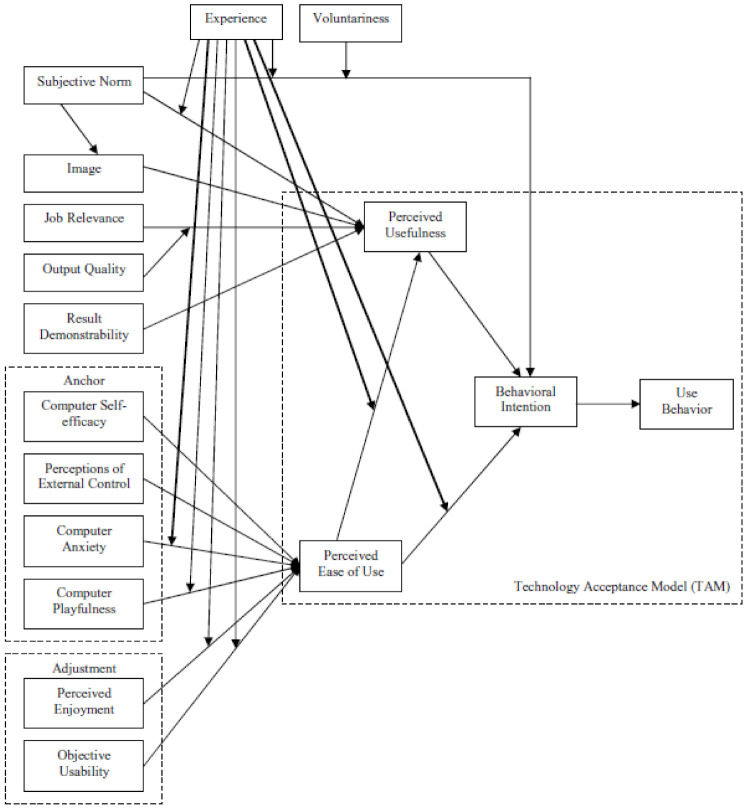
The TAM-3 (The Technology Acceptance Model 3) model. Retrieved from Venkatesh V, Bala H. Technology Acceptance Model 3 and a Research Agenda on Interventions. *Decision Sciences*. **2008**, *39*, 273–315. doi:10.1111/j.1540-5915.2008.00192.x.

The Technology Acceptance Model 3 (TAM-3, shown in Figure 2) and its predecessors have been used to describe the uptake of health care technologies in more than 30 countries [28,29]. The model postulates that perceived usefulness and perceived ease of use shape intention to use technologies and, ultimately, actual use behaviors [30,31]. Systematic reviews have shown that TAM constructs of perceived usefulness of technology and perceived ease of use are the most frequently cited facilitators for adoption of information and communication technologies and mHealth technologies by health care workers [13,32]. Hence, TAM-3 serves as an appropriate conceptual framework to explore and understand factors influencing providers’ use of the ASMAN platform. TAM-3 expanded on earlier versions of the model by incorporating social norms, human and social change processes, and adding additional determinants of perceived ease of use and perceived usefulness [28,31]. In India, the TAM model has been used and adapted to determine the factors of acceptance of health information technology by outpatients in a private-sector hospital [33]; to assess adoption of health information technology by rural healthcare workers [34]; and to model acceptance and usage of a health management information system among workers in a rural healthcare system [35], but it has not been applied to any technologies designed to improve quality of care.

### 2.2. Study Design and Selection of Health Facilities

We conducted qualitative in-depth interviews with health service providers in 12 selected public health facilities where the ASMAN program had been rolled out, including six of 39 ASMAN facilities in Madhya Pradesh state and six of 42 ASMAN facilities in Rajasthan state. We ranked all ASMAN facilities by the percentage of key data fields (identified in accordance with government reporting requirements), completed in the ASMAN application during the six months prior to initiation of data collection (July–December 2019). Within each state’s ranked list, we designated the top quartile as “high-utilization” and the bottom quartile as “low-utilization” facilities to enable comparisons between facilities that were taking full advantage of ASMAN and those where use was lagging. To ensure representation of different states and facility types (community health centers (CHCs) and district/subdistrict hospitals), we purposively identified three facilities from each utilization group in each state (Table 1) using maximum variation sampling. Program officers who are familiar with facility utilization patterns guided this purposive identification process by appraising aspects of ASMAN adoption that were not quantifiable using existing monitoring indicators (e.g., real-time data entry and effective use of ASMAN alerts).

### 2.3. Study Respondents

At each study facility, one medical officer, the labor room supervisor, and two staff nurses were recruited to participate in the study, for a total planned sample size of 48 in-depth interviews, guided by the recommendation that approximately 12 in-depth interviews per stakeholder group are required to reach data saturation [36]. Eligibility criteria included ASMAN training and at least six months experience using ASMAN technologies during regular service provision. In facilities with multiple eligible respondents, the individuals in each cadre who had used ASMAN the longest were selected.

### 2.4. Data Collection

Data collection took place from January to June 2020. All interviews were conducted by three India-based co-investigators of this study who were public health professionals familiar with the ASMAN intervention and had prior experience conducting in-depth interviews. Each interview lasted for 45–50 min. 

Semi-structured interview guides were developed covering topics such as: perceptions of Dakshata training; use of and feedback about specific ASMAN components (e.g., case sheets, gamification, support center); perceptions of the ease of use and usefulness of ASMAN technology, including workload impact; and recommendations for changes to the ASMAN application. Separate interview guides were created for staff nurses, medical officers, and labor room supervisors to reflect their different roles and responsibilities. Questions were open-ended, with appropriate probes to solicit rich accounts of the perceptions and experiences of respondents. Prior to data collection, the interview guides were pretested with two members of each stakeholder group (six total interviews) at two ASMAN facilities in Rajasthan state that were not part of the study sample. Based on the results of the pretesting, the interview guides were refined to ensure appropriate framing, wording, and sequencing of questions. 

A national lockdown was imposed on 24 March 2020 in response to the Covid-19 pandemic. Before the lockdown, 27 in-person in-depth interviews were conducted in-person in private areas within health facilities. Prior to in-person interviews, program staff contacted potential study respondents by phone, informed them about the study, and scheduled interviews. An interviewer then visited each interested respondent at his/her health facility and explained the study objectives. If a provider agreed to participate, the interviewer obtained the respondent’s written informed consent prior to the interview. Privacy and confidentiality were assured during the consent process.

After the lockdown, interviewers completed an additional 17 interviews by phone with respondents from seven facilities (four in Rajasthan and three in Madhya Pradesh). Whether in-person or by phone, interviewers built rapport with respondents by sharing personal background and initiating informal conversation before beginning the in-depth interviews. For phone interviews, oral consent, which was audio recorded, was obtained from respondents prior to the interview. Respondents were asked at the beginning of the call whether they were comfortable and had sufficient privacy at that time for participating in the interview. Respondents were given the option to reschedule the interview if they lacked comfort or privacy at the time of the call. 

All interviews were conducted in Hindi and audio-recorded, lasting 40 min on average. During the interviews, interviewers took detailed hand-written notes and debriefed weekly with the study team in India and the US to identify emerging themes. 

### 2.5. Data Management and Confidentiality

Data were stored in a password-protected hard disk kept in a locked cabinet at the Jhpiego India office. All electronic data, including digital recordings of the interviews and oral consent, were stored as password-protected audio files. Audio files were assigned a unique ID stripped of personally identifiable information. Transcripts of these recordings followed a file naming scheme that differentiated between cadres of health providers, facility type, and ASMAN utilization level, but contained no personally identifiable information. Audio recordings were destroyed after completion of transcription.

### 2.6. Data Analysis

We employed a manifest qualitative content analysis approach to analyze interview transcripts. Using a structured approach to team-based coding developed by MacQueen et al. [37], we created an initial codebook with predetermined codes based on components of the TAM-3 model. This approach allowed us to develop and refine a nested codebook composed of top-level codes and sub-codes with input from all team members [37]. Although the analysis was largely deductive, we allowed for the inclusion of emergent codes, as prior studies have shown the TAM-3 model may not account for all variation in uptake of technology, and many have adapted the TAM [8,38,39].

Four private qualitative research experts were hired to transcribe and translate the interviews into English. One of the co-authors crosschecked each transcript against the original recording to ensure quality, then uploaded transcripts to Dedoose, a web-based qualitative data analysis software application [40]. Each transcript was coded by concept as the unit of analysis, first by top-level codes, then by subcodes. Two co-investigators with qualitative research training based in the United States coded all the transcripts; these co-investigators had also trained the data collection team and supervised the data collection process. To ensure interrater reliability, all codes with less than 85% agreement were discussed and revised by the two coders. All co-authors were involved in interpretation of results.

### 2.7. Ethical Considerations

We received ethical approval for this study from the Institutional Review Board (IRB) (IRB no. 10028) of the Johns Hopkins Bloomberg School of Public Health in the United States and the Sigma IRB (IRB no. 10065/IRB/19–20) in New Delhi, India. Additionally, permission to conduct the study was obtained from both state governments. 

## 3. Results 

We were unable to interview three medical officers and one staff nurse due to their lack of availability for interviews. We interviewed a total of 44 providers. Details about the respondents are given in Table 2 below. 

**Figure 3:**
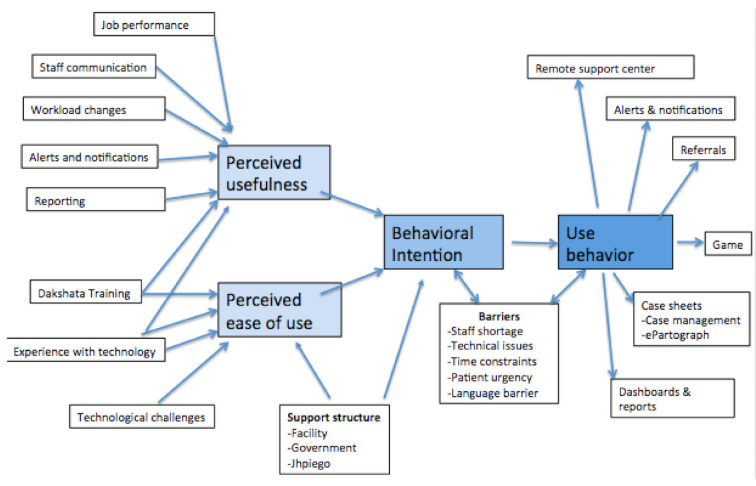
Adapted TAM-3 (The Technology Acceptance Model 3) based on results from qualitative findings related to the uptake of ASMAN.

As described above in the methods section, the TAM-3 shaped our interview guides and data analysis. We grouped our findings based on the concepts and sub-concepts of the TAM-3, and added sub-concepts that emerged from the data to create an adapted version of the model shown in Figure 3. Only major differences in responses per provider cadre were reported in this manuscript. 

### 3.1. Perceived Ease of Use 

#### 3.1.1. Experience with Technology

Before the introduction of ASMAN, many respondents could conduct basic internet searches, use WhatsApp, or use Facebook, but few were familiar with electronic clinical decision-making. Learning to operate new technology was motivating for respondents. They reported ASMAN technology was easy to use overall and easier and faster than hand-written registers.

*Initially we were a bit hesitant to use this application but after training, using it seemed very easy, just like using a mobile phone… We can get all required information about a patient with this application.*

—Labor Room Supervisor, low-utilization CHC

Respondents found it easier to locate electronically stored records and information in the ASMAN platform than paper records. Many labor room supervisors noted that ASMAN allowed for safer record keeping than paper records. Electronic SCC checklists were also perceived as easy to use. The minority of respondents initially frustrated with ASMAN tablet use reported being easily trained by project staff and providers from the same facility. Respondents who did not receive ASMAN training were trained by other providers; all agreed this informal training was sufficient because ASMAN was so user-friendly. Even staff with little previous technological literacy learned to navigate the ASMAN platform.

*Initially operating this application was a big deal for some staff, like one elderly staff person was there who had never used a smartphone… She does not know how to write properly, but she is an expert at using this application.*

—Medical Officer, high-utilization subdistrict hospital

#### 3.1.2. Technological Challenges

Respondents stated that internet connectivity issues and tablets freezing caused the greatest difficulties in using the ASMAN digital platform. Several respondents reported repeated data loss when playing games, disincentivizing game use. Offline data entry suffered from occasional bugs, resulting in double entries or data loss when synced online. Database inflexibility affected some respondents: providers reported being unable to change incorrect entries using the tablet and having to call program staff to make changes, but appreciated that program staff were readily available and corrected errors quickly.

### 3.2. Perceived Usefulness

#### 3.2.1. Dakshata Training

Although Dakshata training was separate from the ASMAN platform, because Dakshata built essential clinical skills it was seen as an important prerequisite to using the ASMAN platform correctly and carrying out clinical recommendations. This was highlighted by respondents who did not attend Dakshata training prior to using ASMAN:

*[T]hey had explained in detail about dosages to be given, there was detailed discussion on PPH [postpartum hemorrhage]. If I had attended, I would have known all that so training is really beneficial.*

—Staff Nurse, high-utilization CHC

Respondents reported universally positive experiences with Dakshata. As a result of the training, they began filling in data more systematically and said their knowledge of correct peripartum practices had greatly improved. Providers said the Dakshata training gave them the necessary knowledge and skills needed to complete fields in the ASMAN digital platform, enhancing perceived ease of use.

#### 3.2.2. Workload Changes

Respondents’ estimates of how their workload changed with the introduction of ASMAN varied. At facilities that continued to use written registers as well as the digital platform, respondents reported increased workload, as nurses had to do double documentation.

*Our workload has increased… We are managing detailed information in the application, as well as in the register, which is time-consuming. If all documentation were restricted only to this application, then it would reduce our workload.*

—Staff Nurse, low-utilization CHC

In facilities that removed written registers, workload decreased as documentation in ASMAN proved less time-consuming and many functions were automated, including e-partograph, discharge slips, and monthly reports. Labor room supervisors particularly found ASMAN to be useful for report generation. But multiple respondents noted that while documentation speed was faster, ASMAN required more time for a thorough intake because it prompted for additional information, such as a more detailed history and physical on admission. Even so, respondents stated this work was not burdensome, given perceived improvements in job performance.

#### 3.2.3. Job Performance

Respondents felt ASMAN improved their ability to take a complete history and physical exam, identify high-risk patients, manage cases confidently, facilitate provider communication, improve reporting processes, and ensure continuity of care for referral patients. Because ASMAN routinized care by requiring information to be entered systematically, or “step-by-step,” human error was reduced.

*Earlier we were not filling the details of everything that was happening in reality during the delivery process… we used to forget some things. But in the app, all the points and fields cover the whole process of delivery, from admission to discharge… we are doing it more carefully now.*

—Staff Nurse, low-utilization CHC

ASMAN also facilitated timely referrals.

*Now we become alert like if there is prolonged labor above 12 h, there is no progress, dilatation is not exceeding more than three fingers, and we also see level of the pains, etc. so we refer the patient.*

—Labor Room Supervisor, high-utilization CHC

Respondents expressed increased confidence in clinical skills among staff using the ASMAN tablet, and the management alerts empowered staff nurses to manage more complicated scenarios independently.

*No timely monitoring was done [before ASMAN] because there was no requirement. High-risk cases used to be referred mostly because nobody called the remote support center. Without timely monitoring, then high-risk cases like obstructed labor or prolonged labor will go wrong. Maternal deaths and stillbirths were happening more often back then… ASMAN has made us more disciplined. Now we know the proper way to manage the patient. It has improved staff knowledge.*

—Labor Room Supervisor, high-utilization CHC

Notifications also improved provider communication and enabled timely discussion with medical officers. Many medical officers and specialists loaded the ASMAN application on their phones and contacted staff nurses with patient care recommendations upon receiving a high-risk notification.

*Sometimes, upon getting a high-risk alert, they [medical officers/specialists] tell us directly to refer the patient. Like if they get an alert of low hemoglobin (which is 7 gm), then they call us and tell us the condition of the patient and instruct us to refer the patient to a higher facility.*

—Labor Room Supervisor, high-utilization CHC

Respondents appreciated how ASMAN facilitated hand-offs to incoming staff during shift changes by displaying patients in the labor and postnatal ward and medication schedules. ASMAN effectively reduced transition time between shifts and facilitated continuity of care. Respondents at all facilities agreed that preparation of digital monthly reports and discharge slips was much faster than compiling handwritten reports. Several respondents mentioned that electronic files were easier to access, less prone to damage, and more permanent than paper files. Staff also used these reports to determine causes of problems when initiating quality improvement processes.

#### 3.2.4. Improved Outcomes

Respondents perceived improvements in peripartum outcomes and related metrics at their facilities after the introduction of ASMAN, including decreases in obstetric complications and stillbirth rates and increases in timely referrals.

*We have reduced referrals, we have managed high-risk cases, and we have also stopped stillbirths by referring on time.*

—Labor Room Supervisor, high-utilization CHC

Additionally, respondents noted that ASMAN’s permanent record served as tangible evidence of their work. If problems arose, it was on record who attended a delivery, enhancing accountability. Some facilities monitored individual completion rates for fields in the ASMAN tablet, which several respondents perceived as a benefit.

*We can show our work to the doctors, directors, and everyone. You were not able to show your work to others via paperwork before. Now we can show that we are doing well and get appreciation.*

—Labor Room Supervisor, high-utilization CHC

### 3.3. Behavioral Intentions

Providers generally felt it was their duty to use the ASMAN application and appreciated its usefulness for improving their job performance and care of patients. They intended to complete all the fields but were not always able to do so.

*[Using ASMAN] is our duty and we must do it for the betterment of the patient… But surely everything would be smoother if we had more staff and ample time.*

—Labor Room Supervisor, low-utilization CHC

One staff nurse noted that staff shortages prevented her from completing all of the fields, but acknowledged they were all necessary, a sentiment many others echoed. Providers noted the following barriers that impeded their use of ASMAN:

1. *Staff shortages:* Well over half of respondents (60%) said staff shortages posed a huge problem for real-time data entry. Often limited to one nurse per shift, respondents said it was impossible to enter data while providing patient care.

*We are fully satisfied [with ASMAN] but our main problem is shortage of staff. It is very difficult for a single staff person to handle 8–10 deliveries per night. We do around 500 deliveries here per month.*

—Staff Nurse, low-utilization district hospital

2. *Patient urgency:* Urgent patient needs often delayed data entry, especially amongst staff nurses.

*Seventy percent of the delivery cases come in full dilatation; in such cases we only complete admission and the rest of the entries are done later. In such cases, we have to manage the patient first.*

—Staff Nurse, low-utilization CHC

3. *Time constraints:* High caseloads and staff shortages limited the time available to use certain features of the application, notably the game. Data entry was also compromised. Medical officers and labor room supervisors cited time constraints as a barrier more frequently than staff nurses.

*Sometimes when I get busy, I forget to enter some data. I do not intentionally omit anything but being only [a nurse at the labor room], I have to handle everything from the outpatient department to attending deliveries, emergency cases, everything.*

—Labor Room Supervisor, low-utilization CHC

4. *Language barriers:* One labor room supervisor noted that some of her staff felt uncomfortable playing the game in ASMAN because there was no Hindi version. Two other respondents also noted the application was in English, a potential barrier for some staff.

### 3.4. Use Behavior

#### 3.4.1. Case Management

Respondents noted improved attention to postnatal care and monitoring, although this section was the most difficult to fill out in real time. All staff nurses used alerts of high-risk situations, such as elevated blood pressure or elevated temperature, to take recommended actions or solicit a doctor’s advice. Alerts were used to ensure timely referrals in cases of prolonged labor and to inform appropriate monitoring intervals in cases of postpartum hemorrhage.

Medical officers and specialists who had the ASMAN application on their phones used the notifications to communicate recommended treatment plans to staff or as an alert to go see a patient themselves.

*Alerts are very helpful… The patient came with a normotensive BP [blood pressure]… After the delivery, I went home. Suddenly the BP of the patient rose, I got the alert, and I came immediately.*

—Medical Officer, high-utilization subdistrict hospital

Due to technological constraints, some medical officers did not use ASMAN alerts and notifications, which their staff saw as a potential hindrance. Some staff were unaware that ASMAN had the ability to send alerts on the doctor’s phone.

*Such a function [alerts] is not in operation here. Our doctor madam has never told us about this alert notification. If this function is there, then it will help us by keeping us alert about any complicating situation.*

—Labor Room Supervisor, low-utilization CHC

The e-partograph had mixed use in facilities. It was perceived as helpful in managing patients and easier to complete than paper partographs, yet faced some of the most significant potential barriers related to patient urgency and shortage of staff and time.

Lower-level facilities used the ASMAN platform to create referral slips. Respondents at higher-level facilities, which received ASMAN-facilitated referrals, reported that they could use the details in ASMAN to anticipate a referred woman’s condition and prepare necessary staff and equipment prior to her arrival.

*Now, when a referral patient is coming and she is from a referral facility, then we can know in advance that she is coming and we prepare accordingly. I remember… [one patient] had postpartum hemorrhage and we noted in the ASMAN app that she was having postpartum hemorrhage, we then checked her other parameters and those were also in critical condition, so we arranged blood transfusions and everything else in advance and when she came everything was ready. When she reached us, she had a cervical tear, so we transfused blood and repaired the tear. Then the patient stabilized… Earlier, when we were doing everything offline, we would never know that such a patient was coming.*

—Staff Nurse, low-utilization district hospital

#### 3.4.2. Dashboards and Reports

Dashboard and reporting features were used at all facilities to help with monthly reporting and reporting of key performance indicators. Several respondents said these reports supported internal quality improvement, allowing assessment of trends in stillbirths, discussion of factors contributing to stillbirths, and brainstorming ways to improve outcomes. Staff at a few facilities used the dashboards to obtain individual records for patients who returned to the facility without their discharge slips.

#### 3.4.3. ASMAN Complication Management Game

The gamification component of ASMAN was underutilized, which respondents attributed to shortages of staff and time, given that they had to prioritize patient care and so were unable to use games for learning purpose. In facilities where the ASMAN game was used, respondents considered it helpful in prioritizing how to manage different scenarios.

*In the game, there are different complicated scenarios about high-risk patients, and you have to determine what to do as a first priority… As you complete all the levels, you get ideas from the games about what is first priority, what should be second, and what should be third. It is very useful for case management.*

—Staff Nurse, high-utilization subdistrict hospital

#### 3.4.4. Remote Support Center

The remote support center was infrequently used. A few respondents felt that it was unnecessary because ASMAN had improved their ability to manage or avoid complications with timely referral and that it improved case management. However, the remote support center was appreciated by the few respondents who consulted it.

*…Once there was a case of severe postpartum hemorrhage, her uterus was not contracting and she was bleeding profusely, we were unable to even refer her to somewhere else, so we called the call center. They explained emergency management and we were able to stop the bleeding. Then we referred her on time.*

—Staff Nurse, high-utilization CHC

Another respondent appreciated the availability of the remote support center during a doctors’ strike, as support center advice facilitated effective complication management without a doctor onsite.

### 3.5. Differences Between High-Utilization and Low-Utilization Facilities

There were some differences in respondents’ perceptions at facilities with the highest versus lowest completion rates for the ASMAN application. Compared with respondents at high-utilization facilities, respondents at low-utilization facilities more often reported barriers to using ASMAN, including staff shortages, time constraints, and patient urgency. These barriers were cited as reasons for respondents’ inability to complete all data fields, add data real-time, or use the game for learning. Technical issues like data connectivity problems were also more often reported at low- than high-utilization facilities.

Respondents at high-utilization facilities reported the perceived usefulness of ASMAN more often than respondents at low-utilization facilities, including for improved histories and physicals, systematic reporting and monitoring, report preparation, safe record-keeping, case management, quality improvement, and the discharge process. Respondents at high-utilization facilities also were more likely to ascribe improved outcomes to ASMAN, including reductions in complications, timeliness of care, efficient handovers, and timely referrals. More medical officers at high- than low-utilization facilities reported using ASMAN on their phone to proactively respond to alerts while on duty.

## 4. Discussion

Respondents across sites and provider cadres uniformly agreed that ASMAN was useful in caring for laboring women and helped with reporting requirements as well as quality assurance activities. ASMAN facilitated a more systematic approach to intrapartum care, enabling providers to perform comprehensive history taking and physical exams step by step, rather than in a more piecemeal fashion. Respondents viewed alerts and guidance as valuable in helping to identify and manage obstetric complications and to prompt more timely referrals. The few who used the support center also appreciated its assistance. Although many respondents were generally familiar with technology, few had prior experience with using it in a clinical setting. Nevertheless, most found ASMAN user-friendly, and even those with little previous technological experience found it easy to use with basic training.

Using the TAM-3 model to guide interviews and interpret the findings allowed us to identify a constellation of factors that shaped the perceived ease of use and perceived usefulness of ASMAN. Our adapted TAM-3 model captured and explained many dimensions of reported respondent behavior, including shifting workflow and job performance. Health providers at high-utilization facilities perceived greater usefulness of ASMAN than health providers at low-utilization facilities, and associated ASMAN with higher-quality documentation as well as a positive impact on maternal and neonatal outcomes. However, the most pronounced barriers to ASMAN use were structural in nature, falling outside of the TAM-3, and were reported by health providers at low-utilization facilities more frequently than health providers at high-utilization facilities. These structural barriers threaten the long-term impact, sustainability, and scalability of ASMAN and similar provider-facing mHealth intervention packages.

### 4.1. Perceived Ease of Use

According to systematic reviews, perceived ease of use is one of the strongest predictors of technology acceptance [41,42]. As our adapted TAM-3 model predicted, support from ASMAN program and the support center, while rarely needed, enhanced respondents’ perceptions of ease of use and facilitated their ability to address problems in a timely manner.

Respondents found the ASMAN technology so easy to use that human error in data entry was actually reduced, and they were able to generate more accurate monthly reports. Training may further amplify this advantage, as studies that have evaluated ongoing eCDSS training for new staff have shown reductions in data entry errors [43,44,45]. Respondents commonly reported that ASMAN platform use resulted in improved history taking and physical examination, which is consistent with other studies showing that use of eCDSSs can improve quality of clinical documentation [46,47,48], although the evidence from LMICs has been more mixed [49,50,51].

Lack of technological proficiency can hinder uptake and utilization of technology [47]; indeed, in the original TAM-3, technology literacy and general competence are prerequisites for technology acceptance [52]. However, we found that previous experience with technology was not a major factor influencing ASMAN platform uptake.

In addition to technological training, clinical training is essential to use the ASMAN apps effectively. In our study, participants received Dakshata clinical training prior to the introduction of ASMAN. Respondents from all facilities viewed the clinical training as crucial to improving providers’ adherence to key clinical practices and use of the ASMAN platform. Although most participants received direct onsite training from ASMAN program staff on how to use ASMAN, the few labor room supervisors or staff nurses who received ASMAN training from other providers felt it was sufficient because ASMAN was so user-friendly. Additionally, regular supervision visits were provided to understand and address clinical and technological challenges faced by providers while using ASMAN application.

### 4.2. Perceived Usefulness

The potential of eCDSSs to improve quality of care is well-documented [47,49]. When quality of care improves, productivity increases. According to the TAM-3 model, result demonstrability (experiencing concrete benefits from using a technology) increases perceived usefulness, which ultimately encourages continued use [31].

There was consensus among respondents that using ASMAN had increased providers’ ability to identify and manage complications, thus reducing overall referral rates, but ensuring timely referrals when necessary. The built-in alerts and notifications also serve as a form of in-service training. The effectiveness of alerts to support eCDSSs have been documented in numerous other studies, including a pilot of eCDSSs in six rural maternal care units in Burkina Faso, where providers credited guidance and built-in alerts with improvements in knowledge; improved symptom recognition helped providers identify problems earlier and prevented adverse outcomes [49]. While there is some concern that “alert fatigue” from excessive reminders could cause decreased use [53,54], health care providers in our study did not report this effect.

The utilization of the e-partograph was varied, with providers at some facilities reporting that staff shortages, high caseloads, and patient urgency created substantial barriers to its use. Similar findings have been reported in Africa. In Ethiopia, health care providers recognized that the e-partograph could reduce maternal and neonatal mortality, but staff shortages, lack of skills/competence, and lack of motivation were mentioned as barriers for partograph utilization [55]. Studies from Kenya and Tanzania found that even where e-partograph is acceptable and has high perceived ease of use, its feasibility in settings with high delivery load, staff shortages, and lack of infrastructure is limited [56,57].

Enhanced workflow has also been documented as a facilitating factor for technology adoption [9,58]. Some respondents felt ASMAN had streamlined their workflow by automating many functions and eliminating multiple registers. However, others complained that ASMAN had increased their workload, often due to requirements for double documentation (handwritten and electronic). A study conducted in maternity care in rural Tanzania and Ghana reported a perceived increase in workload and time required for double data entry as principal barriers for technology adoption [58]. In a study from rural maternal care sites in Burkina Faso, double data entry added approximately 30–40 min of extra workload per patient [49]. Requirements to keep paper registries for verification, audit, or backup in case of unreliable electricity and internet access are understandable as technology is introduced and during a pilot phase. However, this should change as health systems become more comfortable with technology and access becomes more reliable. Sutton et al. warn of the potential of a new eCDSS to disrupt existing workflows if its use is external to other databases or does not fit providers’ regular data documentation routines [46]. Reducing the burden of double documentation by retiring handwritten registers would greatly reduce barriers to use and improve the likelihood of successful scale-up.

Respondents also attributed some increase in workload to more comprehensive data collection prompted by ASMAN, resulting in more thorough assessments of each patient. They believed their enhanced ability and confidence in managing complications would improve peripartum maternal and neonatal outcomes. TAM-3 predicts that improved job performance due to ASMAN (another example of result demonstrability) should enhance its perceived usefulness.

### 4.3. Barriers to ASMAN Use Behavior

Technological challenges including internet connectivity problems, frozen apps, and data loss limited use of ASMAN. Although the same issues have hampered mHealth platform use in studies from Burkina Faso, Ghana, and Tanzania [49,58], these structural barriers largely fall outside the TAM-3 model. Some respondents reported dissatisfaction with inflexible data entry processes in ASMAN, including the difficulty of correcting data entry errors. Systematic reviews have identified a lack of system flexibility as a factor that adversely affects adoption of technology [41,47]. Going forward, adaptive interface and database modifications based on health providers’ feedback may improve ease of use—and data quality—while reducing reliance on project staff to make corrections.

The most underutilized component of ASMAN was the complication management game. Despite the potential of “serious games” and simulations, they have been rarely employed in obstetrics [59]. Gamification has been increasing in many fields and has been associated with improved provider knowledge and skills [60].

Several systems-level barriers that can create workload and workflow issues, which may affect acceptance of new technologies like ASMAN, were also identified. These include staff shortages and turnover; patient urgency limiting real-time data entry; and time constraints. Numerous studies and reviews from LMICs have also noted challenges with staff shortages and turnover [49,58,61]. Importantly, these structural limitations are not a weakness intrinsic to ASMAN design or performance. These structural barriers are common within health systems and may not be easily overcome; however, reducing the double documentation burden, ensuring integration into existing systems, and facilitating easy data entry would help mitigate their impact.

### 4.4. Sustainability Considerations

Despite high acceptability of mHealth interventions, most fail to expand beyond the pilot phase [62,63,64,65], primarily because of sustainability challenges. A majority of health projects rely on donor support and are not fully integrated into the main health system [62]. Moreover, insufficient technical expertise and manpower to support health care providers is another recognized challenge for mHealth sustainability and scalability [63]. However, ASMAN donors, partners, and the governments of both states have worked to ensure sustainability of the ASMAN program by preparing a detailed transition plan. Trainings have been conducted for master trainers, the ASMAN application has been transitioned to states’ servers, and ASMAN fields have been integrated into existing government databases. Additionally, “light-touch” technical assistance and support center functionality is available. Advocacy with both state governments has secured funds for scale-up. Further research will be required to ascertain whether these sustainability efforts will support successful expansion of ASMAN technology, and how learnings from sustainability efforts can be applied to similar programs elsewhere.

### 4.5. Strengths and Limitations

This study provides rich insights and multiple perspectives on the use of a digital mHealth platform to improve the quality of peripartum care in public health facilities in India. The incorporation of a validated and widely accepted theory on technology acceptance contributed to the rigor of the study. Study limitations include the categorization of facilities into “high-utilization” and “low-utilization” groups based on a proxy indicator of data field completion rates because other aspects of utilization were not quantifiable. The selection of individual respondents with the most experience using ASMAN at all facilities may have biased the sample toward higher perceived ease of use. In addition, the COVID-19 pandemic required us to conduct one-third of the in-depth interviews remotely, which complicated rapport-building and may have adversely affected data quality. The Jhpiego affiliation of the study team may have led respondents to give socially desirable answers, although we ensured that no members of the program team were present during interviews and encouraged frank responses. Lastly, this study evaluated perceptions rather than actual use behavior, and we were unable to evaluate the impact of ASMAN on the quality of care provided or on subsequent maternal and neonatal outcomes. Despite these limitations, our findings regarding the tremendous potential of ASMAN to improve quality of care, its high acceptability, and the structural and technological barriers that hampered its ideal implementation are likely transferable to the planning process for similar mHealth interventions in India and globally.

## 5. Conclusions

Significant efforts and investments have been made to ensure that the ASMAN platform is user-friendly, effective, and sustainable. While perceived ease of use, perceived usefulness, and general acceptance of ASMAN were high, respondents reported several barriers to its ideal implementation and eventual scale-up, including staff shortages, time constraints, patient urgency, and problems with connectivity and software functionality. The potential for digital mHealth platforms including eCDSSs to burden—rather than streamline—documentation and reporting, particularly during transition periods, should be anticipated. Future studies are needed to address the impact and scalability of multi-component and user-friendly mHealth platforms like ASMAN that collect high-quality, real-time data during the peripartum period on data-driven decision-making and consequent maternal and neonatal outcomes.

## Figures and Tables

**Table 1:** Number of facilities in study sample by state, type of facility, and utilization group.

State	Type of Health Facility	Utilization of ASMAN (Based on % of Fields Completed)	
		High	Low
Madhya Pradesh	District or subdistrict hospital	1	1
	Community health center (CHC)	2	2
Rajasthan	District or subdistrict hospital	2	1
	Community health center (CHC)	1	2
Total		6	6

**Table 2:** Characteristics of respondents.

Characteristics of Respondents	Madhya Pradesh	Rajasthan	Total
Level of facility			
Community health centers	14	12	26
Subdistrict/district hospitals	6	12	18
Cadre of health provider			
Medical officers	3	6	9
Labor room supervisors	3	7	10
Staff nurse	13	12	25
Age of providers			
Average age	33	42	38
Work experience			
Median work time in the same facility (years)	4	3	5
Median total work experience (years)	7	6	10
Total	20	24	44
